# Towards achievement of Sustainable Development Goal 3: multilevel analyses of demographic and health survey data on health insurance coverage and maternal healthcare utilisation in sub-Saharan Africa

**DOI:** 10.1093/inthealth/ihac017

**Published:** 2022-04-19

**Authors:** Hubert Amu, Richard Gyan Aboagye, Robert Kokou Dowou, Eugene Justine Kongnyuy, Prince Owusu Adoma, Peter Memiah, Elvis Enowbeyang Tarkang, Luchuo Engelbert Bain

**Affiliations:** Department of Population and Behavioural Sciences, School of Public Health, University of Health and Allied Sciences, Hohoe, Ghana; Department of Family and Community Health, School of Public Health, University of Health and Allied Sciences, Hohoe, Ghana; Department of Epidemiology and Biostatistics, School of Public Health, University of Health and Allied Sciences, Hohoe, Ghana; United Nation's Population Fund, UNFPA, Bamako, Mali; Department of Health Administration and Education, Faculty of Science Education, University of Education, Winneba, Ghana; Division of Epidemiology and Prevention: Institute of Human Virology, University of Maryland School of Medicine, Baltimore, Maryland, USA; Department of Population and Behavioural Sciences, School of Public Health, University of Health and Allied Sciences, Hohoe, Ghana; Lincoln International Institute for Rural Health (LIIRH), College of Social Science, University of Lincoln, Lincoln, UK

**Keywords:** health insurance coverage, maternal healthcare utilisation, postnatal care, skilled birth attendance, sub-Saharan Africa, Sustainable Development Goals

## Abstract

**Background:**

Improving maternal health and achieving universal health coverage (UHC) are important expectations in the global Sustainable Development Goals (SDGs) agenda. While health insurance has been shown as effective in the utilisation of maternal healthcare, there is a paucity of literature on this relationship in sub-Saharan Africa (SSA). We examined the relationship between health insurance coverage and maternal healthcare utilisation using demographic and health survey data.

**Methods:**

This was a cross-sectional study of 195 651 women aged 15–49 y from 28 countries in SSA. We adopted bivariable and multivariable analyses comprising χ^2^ test and multilevel binary logistic regression in analysing the data.

**Results:**

The prevalence of maternal healthcare utilisation was 58, 70.6 and 40.7% for antenatal care (ANC), skilled birth attendance (SBA) and postnatal care (PNC), respectively. The prevalence of health insurance coverage was 6.4%. Women covered by health insurance were more likely to utilise ANC (adjusted OR [aOR]=1.48, 95% CI 1.41 to 1.54), SBA (aOR=1.37, 95% CI 1.30 to 1.45) and PNC (aOR=1.42, 95% CI 1.37 to 1.48).

**Conclusion:**

Health insurance coverage was an important predictor of maternal healthcare utilisation in our study. To accelerate progress towards the achievement of SDG 3 targets related to the reduction of maternal mortality and achievement of UHC, countries should adopt interventions to increase maternal insurance coverage, which may lead to higher maternal healthcare access and utilisation during pregnancy.

## Introduction

Improving maternal health outcomes and achieving universal health coverage (UHC) has become part of the global agenda, with substantial emphasis on low- and middle-income countries (LMICs).^1–4^ Maternal healthcare comprises antenatal care (ANC), skilled birth attendance (SBA) and postnatal care (PNC). They are proven services that play a vital role in achieving improved maternal health outcomes.^5^ Inadequate maternal healthcare utilisation, therefore, constitutes a major driver of maternal complications that result in disability and mortality among women in LMICs including sub-Saharan Africa (SSA).^5,6^ More than 800 women, for instance, die during pregnancy, childbirth or within 42 d of termination of pregnancy each day in SSA due to obstetric complications.^7–9^ This militates against the achievement of the Sustainable Development Goals (SDGs) set by the United Nations on 25 September 2015.

The SDGs are a post-2015 development-oriented agenda comprising 17 goals and 169 targets with an overarching aim of achieving global environmental, social and economic development by 2030.^10^ SDG 3 seeks to ensure healthy lives and promote well-being for all ages. Integral to this goal are SDG targets 3.1 and 3.8, which seek to respectively reduce the global maternal mortality ratio to <70 per 100 000 live births and to achieve UHC by 2030.^10^

Direct out-of-pocket payments for maternal healthcare at the point of service constitute a major barrier to the utilisation of maternal healthcare services in SSA.^11–15^ To achieve UHC by eliminating out-of-pocket payments and improving maternal healthcare utilisation as a proxy for reducing maternal deaths, many SSA countries have implemented diverse forms of health insurance, including national, social, private and community-based health insurance schemes.^3,16^

Studies conducted at various country levels in SSA have shown that health insurance improves ANC attendance ^17^, skilled delivery ^18,19^ and PNC among women of reproductive age.^1,20^ There is, however, a paucity of empirical literature at the subregional level on the influence of health insurance coverage on maternal healthcare utilisation. We, therefore, sought to bridge the literature gap by examining the influence of health insurance coverage on maternal healthcare utilisation from 28 countries in SSA countries using the nationally representative demographic and health survey (DHS) data. Also, while many of the previous studies focused on either one or two maternal healthcare variables (ANC, SBA and PNC),^21–24^ we used all of them to achieve a robust outcome variable. The findings from this study could inform policymakers and programme planners regarding the effects of health insurance on maternal healthcare utilisation in SSA.

### Conceptual framework

The study adopted Andersen and Newman's Health Care Utilisation Model as a theoretical framework for the study.^25^ The model is one of the analytical behavioural models proposed to examine diverse factors that influence health service utilisation.^25^ According to the model, utilisation of health services is based on three elements: predisposing, enabling and need for care factors.^25,26^ The predisposing factors consist of demographic characteristics, social structural variables and an individual's basic beliefs, attitudes and knowledge about health services.^26^ Resources available, whether individually or in a community, are examples of enabling factors.^26^ Illnesses, conditions and health statuses that necessitate health services are examples of the need for care factors. The model is a multilevel theory that includes contextual as well as individual-level predictors of healthcare utilisation and this forms the basis for the categorisation of the variables in the study as well as the adoption of the multilevel regression in the analysis.

## Methods

### Data source and study design

The study utilised pooled data from the most recent DHS of 28 countries in SSA. The data were extracted from the women's files (IR Recode) of the selected countries. The DHS is a nationally representative survey usually conducted every 5 y in >85 LMICs.^27^ The survey employed a structured questionnaire to collect data from the respondents on health indicators such as maternal healthcare utilisation.^27^ The survey utilised a cross-sectional design in obtaining the data from the respondents. A two-stage sampling technique was employed to collect data from the respondents. The survey methodology and sampling process have been detailed elsewhere.^28^

In this study, a total of 195 651 women aged 15–49 y were included in the final analysis. Appendix 1 contains the sample distribution per country. We relied on the Strengthening the Reporting of Observational Studies in Epidemiology (STROBE) statement in writing the manuscript.^29^ The dataset is freely available for download at https://dhsprogram.com/ (accessed 28 May 2021).

### Study variables

#### Outcome variables

ANC attendance, SBA and PNC attendance were the outcome variables in this study. From the DHS, the women were asked about the number of ANC visits they made during their recent pregnancies. The responses were recoded as 0–3 ‘No’ and 4 and above ‘Yes’. For SBA, the women were asked ‘Who assisted [NAME] during delivery?’ The response options were regrouped into ‘Traditional Birth Attendant/Others’ and ‘SBA/Health professionals’. Regarding PNC attendance, the women were asked ‘Did [NAME] go for postnatal checks within 2 months?’ The response options to this question were ‘Yes’, ‘No’ and ‘Don't know’. Those who responded ‘Don't know’ were dropped. The recoding and categorisation used in the study were informed by previous literature.^30–34^

### Explanatory variables

The explanatory variables included in the study were based on the review of pertinent literature and centred on the conceptual framework adopted for the study.^30,32^ Also, selection of the variables was based on their availability in the DHS dataset. Based on the constructs of the model, the predisposing variables consisted of the age of the woman, educational level, religion, marital status and parity. The enabling factors consisted of health insurance coverage, mass media, wealth status, place of residence, current working status, permission to go, money for treatment, distance to facility and geographical subregions. The morbidity and mortality to the child or the mother is the need for care factor. The explanatory variables have been further divided into the key explanatory variable and the covariates with their accompanying categorisation, as shown below.

#### Key explanatory variable

The key explanatory variable was health insurance coverage. This variable was assessed in the DHS using the question ‘Are you covered by any health insurance?’ The types of health insurance found in the DHS, which vary per country, include mutual health insurance, national or district health insurance, employer-based health insurance, social security and privately purchased commercial insurance, among others. The response options were ‘No’ and ‘Yes’. Studies have utilised this variable either as a key explanatory variable or as a covariate in determining maternal healthcare utilisation.^1^

### Covariates

A total of 13 variables were studied as covariates in the study. These variables were selected from the predisposing and enabling factors and were categorised into individual- and contextual-level factors, as shown below.

#### Individual-level factors

Maternal age, level of education, marital status, religion, current working status, parity, getting medical help: permission to go, getting medical help: money for treatment and getting medical help: distance to the facility were considered as the individual-level factors. Except for parity, which was recoded as ‘1’, ‘2’, ‘3’ and ‘4 or more’, we utilised the existing coding for the remaining variables as found in the DHS datasets.

#### Contextual-level factors

The contextual variables were wealth index, mass media exposure, place of residence and geographical subregions. From the DHS, the wealth index was coded as ‘poorest’, ‘poorer’, ‘middle’, ‘richer’ and ‘richest’. The place of residence was coded as ‘urban’ and ‘rural’. Exposure to mass media was assessed using three variables (frequency of watching television, frequency of reading newspaper/magazine and frequency of listening to the radio). The response options in each of these variables were ‘not at all’, ‘less than once a week’, ‘at least once a week’ and ‘almost every day’. The response was recoded into ‘No’ (those who responded not at all and less than once a week) and ‘Yes’ (those that responded at least once a week and almost every day). An index variable called mass media exposure was created using the recoded responses from the three variables. Any woman whose response option was ‘Yes’ in each of the variables after the recoding was said to have been exposed to mass media. Those that responded ‘No’ in all three had no exposure to mass media.^33^ Also, the studied countries were further regrouped into the geographical subregions (Eastern, Central, Western and Southern) and used as a contextual-level factor.

### Statistical analyses

Data analysis was carried out using Stata software version 16.0 (Stata Corporation, College Station, TX, USA). The analysis was carried out in three levels. First, percentages were used to present the results of the health insurance coverage and maternal healthcare utilisation (ANC, SBA and PNC) (Table 1). Later, we performed a cross-tabulation to examine the distribution of ANC, SBA and PNC across health insurance coverage, individual- and contextual-level factors, as well as an estimated Pearson's χ^2^ test of independence at p<0.05 to show significant variables (Table 2). The statistically significant variables from the χ^2^ test were placed in the regression model. A multilevel binary logistic regression using five models (Models O–IV) was used to examine the association between health insurance coverage and each of ANC, SBA and PNC, controlling for individual- and contextual-level factors (Tables 3–5).

**Table 1.** Prevalence of maternal healthcare utilisation and health insurance coverage

Countries	ANC	SBA	PNC	Health insurance coverage
Angola	62.4 [59.5–65.1]	53.7 [50.6–56.9]	23.8 [21.9–25.9]	3.8 [3.1–4.6]
Burkina Faso	33.6 [31.8–35.3]	70.8 [68.0–73.5]	81.9 [80.0–83.7]	0.4 [0.3–0.6]
Benin	54.5 [52.1–57.0]	82.0 [79.5–84.4]	19.2 [17.5–21.0]	0.9 [0.6–1.1]
Burundi	49.3 [47.6–50.9]	85.5 [84.1–86.7]	8.5 [7.7–9.5]	24.2 [22.6–25.9]
DR Congo	48.7 [46.3–51.1]	81.2 [78.3–83.8]	18.3 [16.2–20.7]	3.6 [2.7–4.7]
Congo	79.6 [77.4–81.5]	93.4 [92.0–94.6]	56.9 [53.4–60.3]	2.5 [1.9–3.3]
Cote d'Ivoire	45.0 [41.8–48.1]	62.3 [58.3–66.2]	70.3 [67.5–73.0]	2.2 [1.6–3.1]
Cameroon	65.8 [62.6–68.8]	71.4 [67.3–75.2]	28.2 [25.9–30.6]	1.8 [1.4–2.3]
Ethiopia	32.0 [29.4–34.7]	31.1 [27.7–34.7]	8.3 [7.2–9.6]	4.2 [3.1–5.5]
Gabon	78.0 [77.7–82.1]	91.9 [90.3–93.3]	57.3 [53.5–61.0]	43.3 [39.8–46.9]
Ghana	87.7 [85.6–89.6]	76.0 [72.6–79.0]	72.5 [68.8–75.9]	66.8 [64.4–69.0]
Gambia	75.8]69.1–75.8]	14.1 [11.0–18.0]	71.8 [66.5–76.4]	0.6 [0.3–1.3]
Guinea	37.8 [35.2–40.5]	57.0 [53.3–60.5]	35.4 [32.6–38.3]	1.1 [0.7–1.6]
Kenya	58.5 [56.7–60.3]	66.7 [64.9–68.6]	68.0 [66.3–69.6]	17.3 [15.8–19.0]
Comoros	63.1 [58.6–67.4]	85.2 [82.5–87.6]	32.1 [28.0–36.5]	4.5 [3.5–5.8]
Liberia	89.1 [87.4–90.6]	86.2 [83.6–88.4]	24.7 [22.3–27.2]	2.7 [2.0–3.7]
Lesotho	75.0 [72.7–77.2]	79.8 [77.5–81.9]	81.8 [79.7–83.7]	1.4 [0.9–2.1]
Mali	45.6 [42.7–48.5]	73.0 [69.3–76.4]	25.5 [22.9–28.2]	4.7 [3.6–6.1]
Malawi	51.0 [49.6–52.4]	90.3 [89.3–91.3]	44.3 [42.1–46.5]	1.3 [0.8–2.1]
Nigeria	58.3 [56.4–60.2]	48.2 [46.0–50.3]	20.9 [19.7–22.1]	2.2 [1.8–2.6]
Namibia	85.9 [84.2–87.4]	89.8 [88.2–91.2]	54.6 [51.9–57.2]	14.0 [12.1–16.2]
Sierra Leone	90.7 [89.5–91.8]	88.3 [86.5–89.9]	45.6 [42.6–48.6]	3.8 [2.7–5.2]
Senegal	52.1 [49.5–54.8]	65.4 [61.5–69.1]	73.3 [70.9–75.6]	4.6 [3.6–5.7]
Chad	34.1 [31.3–37.0]	36.2 [32.8–39.7]	17.7 [14.8–20.9]	0.9 [0.6–1.4]
Togo	57.6 [55.1–60.1]	61.5 [57.1–65.6]	72.3 [69.6–74.8]	4.1 [3.3–5.1]
Uganda	60.5 [59.0–62.0]	77.2 [75.3–79.0]	22.6 [21.1–24.1]	1.2 [0.9–1.6]
Zambia	64.4 [62.6–66.7]	82.6 [80.6–84.4]	63.3 [61.0–65.5]	1.9 [1.4–2.6]
Zimbabwe	76.0 [74.1–77.9]	80.5 [78.1–82.8]	84.2 [82.1–86.1]	8.8 [7.2–10.7]
**All countries**	**58.0 [57.8–58.2]**	**70.6 [70.4–70.8]**	**40.7 [40.5–40.9]**	**6.4 [6.3–6.6]**

**Table 2.** Bivariable analysis of health insurance coverage and maternal healthcare service utilisation among women in SSA

			ANC		SBA		PNC	
Variables	Weighted N	Weighted %	Yes	p^*^	Yes	p^*^	Yes	p^*^
**Health insurance coverage**				<0.001		<0.001		<0.001
No	183 033	93.6	56.7		69.5		39.8	
Yes	12 618	6.4	76.6		86.7		53.8	
**Maternal age, y**				<0.001		<0.001		<0.001
15–19	14 512	7.4	54.2		72.1		38.2	
20–24	44 148	22.6	58.0		72.8		41.0	
25–29	50 315	25.7	59.1		71.5		40.8	
30–34	39 530	20.2	59.5		70.7		41.9	
35–39	28 532	14.6	58.3		69.1		40.5	
40–44	13 947	7.1	55.5		64.8		39.9	
45–49	4667	2.4	51.8		59.6		37.0	
**Maternal educational level**				<0.001		<0.001		<0.001
No education	74 026	37.8	43.2		53.1		35.8	
Primary	61 045	31.2	57.8		73.5		39.7	
Secondary	52 898	27.1	74.8		88.1		47.2	
Higher	7682	3.9	87.8		96.2		50.7	
**Marital status**				<0.001		<0.001		<0.001
Never married	14 927	7.6	68.3		83.5		44.2	
Married	134 943	69.0	55.2		67.3		41.3	
Cohabiting	31 683	16.2	64.3		76.5		36.0	
Widowed	2765	1.4	58.0		65.5		42.4	
Divorced	3209	1.6	57.9		72.9		45.3	
Separated	8124	4.2	61.1		79.6		39.9	
**Religion**				<0.001		<0.001		<0.001
Christianity	120 620	61.7	63.1		78.2		41.0	
Islamic	66 317	33.9	50.5		58.7		39.5	
African traditional	3424	1.7	39.4		49.2		47.4	
No religion	4246	2.2	45.7		55.7		44.5	
Others	1044	0.5	61.5		74.5		37.0	
**Maternal current working status**				<0.001		<0.001		0.096
No	67 757	34.6	55.6		67.4		40.2	
Yes	127 894	65.4	59.3		72.3		40.9	
**Parity**				<0.001		<0.001		<0.001
1	41 133	21.0	65.0		81.5		44.4	
2	36 970	18.9	61.7		75.6		43.0	
3	31 368	16.0	59.9		72.4		42.4	
4 or more	86 180	44.1	52.4		62.6		37.3	
**Getting medical help for self: permission to go**				<0.001		<0.001		<0.001
Not a major problem	156 234	79.9	60.1		72.3		42.8	
Major problem	39 417	20.1	49.9		63.7		32.1	
**Getting medical help for self: distance to a health facility**				<0.001		<0.001		<0.001
Not a major problem	116 987	59.8	62.7		76.6		42.8	
Major problem	78 664	40.2	51.1		61.6		37.5	
**Getting medical help for self: getting money for treatment**				<0.001		<0.001		<0.001
Not a major problem	86 301	44.1	64.3		76.1		44.0	
Major problem	109 350	55.9	53.1		66.2		38.1	
**Mass media exposure**				<0.001		<0.001		<0.001
No	103 284	52.8	50.0		62.2		33.8	
Yes	92 367	47.2	67.0		80.0		48.4	
**Wealth index**				<0.001		<0.001		<0.001
Poorest	41 748	21.4	44.6		50.5		35.2	
Poorer	41 681	21.3	51.2		60.4		37.8	
Middle	39 369	20.1	57.4		70.7		40.7	
Richer	38 201	19.5	64.7		82.9		43.5	
Richest	34 652	17.7	75.7		93.4		47.5	
**Residence**				<0.001		<0.001		<0.001
Urban	67 892	34.7	72.6		87.1		46.6	
Rural	127 759	65.3	50.3		61.8		37.5	

*p-values obtained from χ^2^ test.

**Table 3.** Fixed and random effects analysis on the association between health insurance coverage and ANC among women in SSA

Variable	Model O	Model I cOR [95% CI]	Model II aOR [95% CI]	Model III aOR [95% CI]	Model IV aOR [95% CI]
**Fixed effect results**					
**Health insurance coverage**					
No		1 [1.00 to 1.00]			1 [1.00 to 1.00]
Yes		2.27*** [2.18 to 2.36]			1.48*** [1.41 to 1.54]
**Maternal age, y**					
15–19			1 [1.00 to 1.00]		1 [1.00 to 1.00]
20–24			1.27*** [1.22 to 1.32]		1.20*** [1.15 to 1.25]
25–29			1.63*** [1.56 to 1.71]		1.43*** [1.37 to 1.50]
30–34			1.97*** [1.87 to 2.07]		1.65*** [1.57 to 1.74]
35–39			2.14*** [2.03 to 2.29]		1.77*** [1.67 to 1.87]
40–44			2.07*** [1.95 to 2.19]		1.71*** [1.61 to 1.82]
45–49			2.10*** [1.95 to 2.26]		1.74*** [1.61 to 1.87]
**Maternal educational level**					
No education			1 [1.00 to 1.00]		1 [1.00 to 1.00]
Primary			1.64*** [1.60 to 1.68]		1.672*** [1.63 to 1.72]
Secondary			3.01*** [2.92 to 3.09]		2.499*** [2.42 to 2.58]
Higher			5.66*** [5.25 to 6.11]		3.970*** [3.67 to 4.30]
**Marital status**					
Never married			1 [1.00 to 1.00]		1 [1.00 to 1.00]
Married			0.89*** [0.85 to 0.92]		0.979 [0.94 to 1.02]
Cohabiting			1.09*** [1.04 to 1.14]		1.185*** [1.13 to 1.24]
Widowed			0.94 [0.86 to 1.02]		1.071 [0.98 to 1.17]
Divorced			0.83*** [0.76 to 0.90]		1.046 [0.96 to 1.14]
Separated			0.89*** [0.84 to 0.98]		1.033 [0.97 to 1.10]
**Religion**					
Christianity			1 [1.00 to 1.00]		1 [1.00 to 1.00]
Islamic			0.94*** [0.92 to 0.96]		0.760*** [0.74 to 0.78]
African traditional			0.65*** [0.60 to 0.69]		0.544*** [0.51 to 0.58]
No religion			0.65*** [0.62 to 0.71]		0.63*** [0.59 to 0.67]
Others			0.86* [0.76 to 0.98]		0.75*** [0.65 to 0.85]
**Maternal current working status**					
No			1 [1.00 to 1.00]		1 [1.00 to 1.00]
Yes			1.17*** [1.15 to 1.20]		1.16*** [1.13 to 1.18]
**Parity**					
1			1 [1.00 to 1.00]		1 [1.00 to 1.00]
2			0.83*** [0.80 to 0.85]		0.84*** [0.81 to 0.87]
3			0.75*** [0.72 to 0.78]		0.79*** [0.76 to 0.82]
4 or more			0.59*** [0.56 to 0.61]		0.67*** [0.64 to 0.69]
**Getting medical help for self: getting money for treatment**					
Not a major problem			1 [1.00 to 1.00]		1 [1.00 to 1.00]
Major problem			0.85*** [0.83 to 0.86]		0.88*** [0.86 to 0.90]
**Getting medical help for self: distance to a health facility**					
Not a major problem			1 [1.00 to 1.00]		1 [1.00 to 1.00]
Major problem			0.81*** [0.80 to 0.83]		0.91*** [0.89 to 0.93]
**Getting medical help for self: permission to go**					
Not a major problem			1 [1.00 to 1.00]		1 [1.00 to 1.00]
Major problem			0.85*** [0.83 to 0.87]		0.86*** [0.84 to 0.88]
**Mass media exposure**					
No				1 [1.00 to 1.00]	1 [1.00 to 1.00]
Yes				1.44*** [1.41 to 1.47]	1.24*** [1.21 to 1.26]
**Wealth index**					
Poorest				1 [1.00 to 1.00]	1 [1.00 to 1.00]
Poorer				1.26*** [1.23 to 1.29]	1.14*** [1.11 to 1.17]
Middle				1.41*** [1.37 to 1.45]	1.20*** [1.16 to 1.23]
Richer				1.59*** [1.54 to 1.64]	1.24*** [1.20 to 1.29]
Richest				2.13*** [2.06 to 2.22]	1.34*** [1.29 to 1.39]
**Place of residence**					
Urban				1 [1.00 to 1.00]	1 [1.00 to 1.00]
Rural				0.66*** [0.64 to 0.68]	0.76*** [0.74 to 0.78]
**Sub-regions**					
Southern				1 [1.00 to 1.00]	1 [1.00 to 1.00]
Central				0.33*** [0.31 to 0.36]	0.44*** [0.41 to 0.47]
East				0.32*** [0.30 to 0.34]	0.42*** [0.39 to 0.45]
West				0.33*** [0.31 to 0.35]	0.65*** [0.60 to 0.69]
**Random effect results**					
PSU variance [95% CI]	0.208 [0.180 to 0.242]	0.203 [0.175 to 0.236]	0.068 [0.057 to 0.081]	0.090 [0.075 to 0.107]	0.052 [0.044 to 0.063]
ICC	0.060	0.058	0.020	0.026	0.016
LR test	1701.41 (<0.001)	1640.48 (<0.001)	820.31 (<0.001)	963.44 (<0.001)	668.96 (<0.001)
Wald χ^2^	Reference	1560.90***	15 408.55***	10 308.00***	18 431.85***
**Model fitness**					
Log-likelihood	−132 761.69	−131 902.55	−124 077.67	−127 127.56	−121 966.8
AIC	265 527.4	263 811.1	248 209.3	254 277.1	244 007.6
N	195 651	195 651	195 651	195 651	195 651
Number of clusters	1611	1611	1611	1611	1611

Abbreviations: AIC, Akaike's information criterion; ICC, intra-class correlation; LR test, likelihood ratio test; PSU, primary sampling unit.

* p<0.05; ** p<0.01; *** p<0.001; 1=reference.

**Table 4.** Fixed and random effects results on the association between health insurance coverage and SBA utilisation among women in SSA

Variable	Model 0	Model I aOR [95% CI]	Model II aOR [95% CI]	Model III aOR [95% CI]	Model IV aOR [95% CI]
**Fixed effect results**					
**Health insurance coverage**					
No		1 [1.00 to 1.00]			1 [1.00 to 1.00]
Yes		2.48*** [2.37 to 2.61]			1.37*** [1.30 to 1.45]
**Maternal age, y**					
15–19			1 [1.00 to 1.00]		1 [1.00 to 1.00]
20–24			1.27*** [1.22 to 1.34]		1.12*** [1.06 to 1.17]
25–29			1.73*** [1.64 to 1.82]		1.32*** [1.24 to 1.39]
30–34			2.17*** [2.05 to 2.31]		1.52*** [1.43 to 1.61]
35–39			2.30*** [2.16 to 2.44]		1.58*** [1.48 to 1.68]
40–44			2.14*** [2.00 to 2.29]		1.49*** [1.39 to 1.60]
45–49			2.02*** [1.86 to 2.20]		1.43*** [1.31 to 1.56]
**Maternal educational level**					
No education			1 [1.00 to 1.00]		1 [1.00 to 1.00]
Primary			2.03*** [1.98 to 2.08]		1.69*** [1.65 to 1.74]
Secondary			4.27*** [4.12 to 4.41]		2.72*** [2.62 to 2.82]
Higher			12.42*** [10.91 to 14.16]		4.25*** [3.71 to 4.87]
**Marital status**					
Never married			1 [1.00 to 1.00]		1 [1.00 to 1.00]
Married			0.97 [0.92 to 1.02]		0.92** [0.87 to 0.97]
Cohabiting			0.98 [0.92 to 1.03]		1.03 [0.98 to 1.09]
Widowed			0.82*** [0.75 to 0.91]		0.81*** [0.74 to 0.90]
Divorced			0.93 [0.85 to 1.03]		0.90* [0.81 to 0.99]
Separated			1.09* [1.01 to 1.17]		1.124** [1.04 to 1.21]
**Religion**					
Christianity			1 [1.00 to 1.00]		1 [1.00 to 1.00]
Islamic			0.62*** [0.61 to 0.64]		0.54*** [0.52 to 0.56]
African traditional			0.46*** [0.42 to 0.49]		0.50*** [0.47 to 0.54]
No religion			0.46*** [0.44 to 0.50]		0.54*** [0.51 to 0.58]
Others			0.72*** [0.62 to 0.83]		0.71*** [0.61 to 0.83]
**Maternal working status**					
No			1 [1.00 to 1.00]		1 [1.00 to 1.00]
Yes			1.30*** [1.27 to 1.33]		1.38*** [1.34 to 1.41]
**Parity**					
1			1 [1.00 to 1.00]		1 [1.00 to 1.00]
2			0.63*** [0.61 to 0.66]		0.67*** [0.64 to 0.69]
3			0.52*** [0.49 to 0.54]		0.57*** [0.55 to 0.60]
4 or more			0.36*** [0.34 to 0.37]		0.46*** [0.44 to 0.48]
**Getting medical help for self: getting money for treatment**					
Not a major problem			1 [1.00 to 1.00]		1 [1.00 to 1.00]
Major problem			0.93*** [0.91 to 0.96]		1.06*** [1.04 to 1.09]
**Getting medical help for self: distance to a health facility**					
Not a major problem			1 [1.00 to 1.00]		1 [1.00 to 1.00]
Major problem			0.56*** [0.54 to 0.57]		0.67*** [0.66 to 0.69]
**Getting medical help for self: permission to go**					
Not a major problem			1 [1.00 to 1.00]		1 [1.00 to 1.00]
Major problem			1.00 [0.97 to 1.02]		1.05** [1.02 to 1.08]
**Mass media exposure**					
No				1 [1.00 to 1.00]	1 [1.00 to 1.00]
Yes				1.35*** [1.32 to 1.38]	1.16*** [1.13 to 1.19]
**Wealth index**					
Poorest				1 [1.00 to 1.00]	1 [1.00 to 1.00]
Poorer				1.54*** [1.50 to 1.59]	1.37*** [1.33 to 1.41]
Middle				2.19*** [2.12 to 2.25]	1.85*** [1.80 to 1.91]
Richer				3.65*** [3.52 to 3.79]	2.93*** [2.81 to 3.04]
Richest				8.25*** [7.81 to 8.72]	5.30*** [5.00 to 5.61]
**Place of residence**					
Urban				1 [1.00 to 1.00]	1 [1.00 to 1.00]
Rural				0.50*** [0.49 to 0.52]	0.588*** [0.57 to 0.61]
**Subregions**					
Southern				1 [1.00 to 1.00]	1 [1.00 to 1.00]
Central				0.44*** [0.41 to 0.48]	0.56*** [0.52 to 0.60]
Eastern				0.76*** [0.70 to 0.81]	1.09* [1.01 to 1.18]
Western				0.34*** [0.32 to 0.37]	0.81*** [0.75 to 0.88]
**Random effect results**					
PSU variance (95% CI)	0.455 [0.405 to 0.511]	0.444 [0.394 to 0.500]	0.507 [0.451 to 0.570]	0.403 [0.358 to 0.454]	0.586 [0.523 to 0.656]
ICC	0.122	0.119	0.133	0.109	0.151
LR Test	4419.42 (<0.001)	4259.72 (<0.001)	3609.25 (<0.001)	3706.37 (<0.001)	3659.86 (<0.001)
Wald χ^2^	Reference	1343.11***	23 309.51***	21 659.98***	28 957.98***
**Model fitness**					
Log-likelihood	−119 099.91	−118 306.93	−104 065.9	−104 484.69	−97 861.306
AIC	238 203.8	236 619.9	208 185.8	208 991.4	195 796.6
N	195 651	195 651	195 651	195 651	195 651
Number of clusters	1611	1611	1611	1611	1611

Abbreviations: AIC, Akaike's information criterion; ICC, intra-class correlation; LR test, likelihood ratio test; PSU, primary sampling unit.

* p<0.05; ** p<0.01; *** p<0.001; 1=reference.

**Table 5.** Fixed and random effects results on the association between health insurance and PNC among women in SSA

	Model 0	Model I cOR [95% CI]	Model II aOR [95% CI]	Model III aOR [95% CI]	Model IV aOR [95% CI]
**Fixed effects results**					
**Health insurance coverage**					
No		1 [1.00 to 1.00]			1 [1.00 to 1.00]
Yes		1.62*** [1.57 to 1.68]			1.42*** [1.37 to 1.48]
**Maternal age, y**					
15–19			1 [1.00 to 1.00]		1 [1.00 to 1.00]
20–24			1.18*** [1.13 to 1.23]		1.09*** [1.05 to 1.14]
25–29			1.35*** [1.29 to 1.41]		1.17*** [1.12 to 1.22]
30–34			1.56*** [1.48 to 1.64]		1.29*** [1.23 to 1.36]
35–39			1.59*** [1.51 to 1.68]		1.30*** [1.23 to 1.37]
40–44			1.62*** [1.53 to 1.72]		1.32*** [1.24 to 1.40]
45–49			1.54*** [1.43 to 1.67]		1.25*** [1.16 to 1.35]
**Maternal educational level**					
No education			1 [1.00 to 1.00]		1 [1.00 to 1.00]
Primary			1.25*** [1.22 to 1.28]		1.30*** [1.27 to 1.33]
Secondary			1.51*** [1.47 to 1.55]		1.42*** [1.38 to 1.47]
Higher			1.42*** [1.35 to 1.51]		1.23*** [1.16 to 1.31]
**Marital status**					
Never married			1 [1.00 to 1.00]		1 [1.00 to 1.00]
Married			1.08*** [1.04 to 1.12]		1.12*** [1.08 to 1.17]
Cohabiting			0.79*** [0.76 to 0.83]		0.91*** [0.87 to 0.95]
Widowed			1.14** [1.05 to 1.24]		1.25*** [1.15 to 1.36]
Divorced			1.21*** [1.11 to 1.31]		1.41*** [1.31 to 1.54]
Separated			0.92** [0.87 to 0.98]		1.09** [1.02 to 1.15]
**Religion**					
Christianity			1 [1.00 to 1.00]		1 [1.00 to 1.00]
Islamic			1.12*** [1.10 to 1.15]		0.89*** [0.87 to 0.92]
African traditional			1.89*** [1.76 to 2.03]		1.50*** [1.40 to 1.61]
No religion			1.35*** [1.27 to 1.44]		1.30*** [1.22 to 1.38]
Others			0.78*** [0.69 to 0.89]		0.61*** [0.53 to 0.70]
**Parity**					
1			1 [1.00 to 1.00]		1 [1.00 to 1.00]
2			0.88*** [0.85 to 0.91]		0.91*** [0.88 to 0.94]
3			0.81*** [0.78 to 0.84]		0.87*** [0.84 to 0.91]
4 or more			0.65*** [0.62 to 0.67]		0.75*** [0.73 to 0.78]
**Getting medical help for self: getting money for treatment**					
Not a major problem			1 [1.00 to 1.00]		1 [1.00 to 1.00]
Major problem			0.97* [0.95 to 0.99]		1.05*** [1.02 to 1.07]
**Getting medical help for self: distance to a health facility**					
Not a major problem			1 [1.00 to 1.00]		1 [1.00 to 1.00]
Major problem			0.97** [0.95 to 0.99]		1.01 [0.99 to 1.04]
**Getting medical help for self: permission to go**					
Not a major problem			1 [1.00 to 1.00]		1 [1.00 to 1.00]
Major problem			0.69*** [0.67 to 0.71]		0.78*** [0.76 to 0.80]
**Mass media exposure**					
No				1 [1.00 to 1.00]	1 [1.00 to 1.00]
Yes				1.65*** [1.62 to 1.68]	1.58*** [1.55 to 1.62]
**Wealth index**					
Poorest				1 [1.00 to 1.00]	1 [1.00 to 1.00]
Poorer				1.04** [1.01 to 1.07]	1.02 [0.99 to 1.05]
Middle				1.03 [1.00 to 1.06]	0.99 [0.96 to 1.02]
Richer				1.01 [0.97 to 1.04]	0.94*** [0.91 to 0.97]
Richest				0.96* [0.92 to 1.00]	0.83*** [0.80 to 0.86]
**Place of residence**					
Urban				1 [1.00 to 1.00]	1 [1.00 to 1.00]
Rural				0.83*** [0.81 to 0.85]	0.87*** [0.84 to 0.89]
**Subregions**					
Southern				1 [1.00 to 1.00]	1 [1.00 to 1.00]
Central				0.24*** [0.22 to 0.25]	0.28*** [0.26 to 0.29]
East				0.44*** [0.41 to 0.46]	0.46*** [0.44 to 0.49]
West				0.52*** [0.50 to 0.55]	0.65*** [0.61 to 0.69]
**Random effect results**					
PSU variance [95% CI]	0.312 [0.275 to 0.354]	0.303 [0.267 to 0.344]	0.283 [0.250 to 0.321]	0.281 [0.247 to 0.319]	0.263 [0.231 to 0.299]
ICC	0.087	0.084	0.079	0.076	0.074
LR Test	3296.38 (<0.001)	3154.26 (<0.001)	3256.73 (<0.001)	2982.80 (<0.001)	2830.50 (<0.001)
Wald χ^2^	Reference	687.91***	4104.77***	7694.94***	9924.79
**Model fitness**					
Log-likelihood	−130 299.81	−129 956.05	−128 175.14	−126 183.84	−124 888.57
AIC	260 603.6	259 918.1	256 402.3	252 389.7	249 849.1
N	195 651	195 651	195 651	195 651	195 651
Number of clusters	1611	1611	1611	1611	1611

Abbreviations: AIC, Akaike's information criterion; ICC, intra-class correlation; LR test, likelihood ratio test; PSU, primary sampling unit.

* p<0.05; ** p<0.01; *** p<0.001; 1=reference.

Model O showed the variance in ANC, SBA and PNC attributed to the clustering of the primary sampling units (PSUs) without health insurance coverage and studied covariates. Model I was fitted to contain health insurance coverage alone. Model II was fitted to contain the individual-level factors. Model III contained the contextual-level factors. Model IV was finally fitted to comprise health insurance coverage, individual- and contextual-level factors. The Stata command ‘melogit’ was used in fitting the five models. Fixed and random effects were included in all five models. The fixed effects represented the relationship between the explanatory variable and/or covariates and the outcome variable, whereas the random effects represented the measure of variation in the outcome variable based on PSU, as measured by intra-cluster correlation (ICC). As a result, the ICC was used to quantify the differences between clusters in the sample used for the analysis. Finally, Akaike's information criterion (AIC) was used to examine the model fitness, or how the various models were fitted with the data. From the models, the one with the least AIC value was selected as the best-fitted model. Thus, the final models (IV) in Tables 3–5 were chosen for forecasting the association between ANC, SBA and PNC and health insurance coverage. All the regression results were presented using crude ORs (cORs) and adjusted ORs (aORs) at a 95% CI. The women's sample weight (v005/1000 000) was applied to the data to cater for the complex nature of the DHS dataset. The Stata command ‘svy’ was used to adjust for the disproportionate sampling and non-response and to improve the generalisability of the findings.

## Results

### Prevalence of maternal healthcare utilisation and health insurance coverage

Table 1 presents the percentage prevalence of maternal healthcare utilisation and health insurance coverage among women in SSA. The overall prevalence of maternal healthcare utilisation among women of reproductive age in SSA was 58, 70.6 and 40.7% for ANC, SBA and PNC, respectively. There were, however, inter-country variations. The highest prevalence of ANC, SBA and PNC utilisation was, for instance, recorded in Sierra Leone (90.7%), Congo (93.4%) and Zimbabwe (84.2%), respectively. The lowest prevalence of ANC, SBA and PNC utilisation was, respectively, recorded in Ethiopia (32%), Gambia (14.1%) and Ethiopia (8.3%). Regarding health insurance coverage, the overall prevalence was 6.4% with country-level variations. While Ghana recorded the highest coverage (66.8%), the lowest was recorded in Burkina Faso (0.4%).

### Bivariable relationship between health insurance coverage and maternal healthcare utilisation

Table 2 presents bivariable results on the relationship between health insurance coverage and maternal healthcare utilisation among women in SSA. We found that health insurance coverage was statistically related to ANC (p<0.001), SBA (p<0.001) and PNC (p<0.001). We also found statistically significant relationships between maternal healthcare utilisation and maternal age, maternal educational level, marital status, religion, parity, permission to access maternal healthcare, distance to a health facility, getting money for treatment, mass media exposure, wealth index and residence.

### Association between health insurance coverage and maternal healthcare utilisation

From Table 3, women covered by health insurance were more likely to utilise ANC (aOR=1.48, 95% CI 1.41 to 1.54). The probability of ANC utilisation among women of reproductive age increased with maternal age, maternal education and wealth index. Women who were cohabiting (aOR=1.19, 95% CI 1.13 to 1.24), currently working (aOR=1.16, 95% CI 1.13 to 1.18) and exposed to the mass media (aOR=1.24, 95% CI 1.21 to 1.26) had higher odds of utilising ANC. On the other hand, multiparous women, who had difficulties getting money for treatment (aOR=0.88, 95% CI 0.86 to 0.90), whose residences were distant from a health facility (aOR=0.91, 95% CI 0.89 to 0.93), had trouble getting permission to access ANC (aOR=0.86, 95% CI 0.84 to 0.88) and resided in rural areas (aOR=0.86, 95% CI 0.84 to 0.88) had lower odds of utilising ANC. No discernible patterns were observed between religion, subregion and ANC utilisation.

### Random effect results for ANC

Results from Table 3, Model O, showed that ANC varies significantly across the clusters O (σ2=0.208, 95% CI 0.180 to 0.242). Model O also revealed that the between-cluster variations were responsible for 6% of the ANC prevalence (ICC=0.060). The between-cluster variation decreased to 2% in the model containing only individual-level variables (Model II), increased to 3% in the model containing contextual-level variables (Model III) and then decreased to about 2% in the model containing all individual- and contextual-level variables. This suggests that the variances across the clusters can explain the differences in the probability of attending ANC. In addition, the AIC value decreased from the empty model (Model O) to the complete model (Model IV). This confirms the final model’s (Model IV) goodness of fit, which was established in the analysis. As a result, Model IV was chosen to estimate the relationship between ANC and health insurance among women.

### Fixed effect results for SBA

From Table 4, women covered by health insurance were more likely to utilise SBA (aOR=1.37, 95% CI 1.30 to 1.45). The probability of SBA utilisation among women of reproductive age increased with maternal education and wealth index. The odds of utilising SBA were highest among women in their late 30s (aOR=1.58, 95% CI 1.48 to 1.68) and separated (aOR=1.12, 95% CI 1.04 to 1.21). Women who were currently working (aOR=1.38, 95% CI 1.34 to 1.41) and exposed to the mass media (aOR=1.16, 95% CI 1.13 to 1.19) had higher odds of utilising SBA. On the contrary, multiparous women, women who had a major problem with the distance to a health facility (aOR=0.67, 95% CI 0.66 to 0.69) and resided in rural areas (aOR=0.59, 95% CI 0.57 to 0.61) had lower odds of utilising ANC. No discernible patterns were observed between religion, subregion and SBA utilisation.

### Random effect results for SBA

In Table 4, the results in Model O indicate that SBA varies across the clusters and this variation was approximately 46% (σ2=0.455, 95% CI 0.405 to 0.511). In the same model, 12% of the prevalence of SBA was associated with the between-cluster variations (ICC=0.122). The between-cluster variations varied significantly across the five models, with Model IV exhibiting the greatest variation (ICC=0.151). This suggests that the variances across the clusters can explain the differences in the probability of attending ANC. Furthermore, the AIC value decreased from the empty model (Model O) to the complete model (Model IV). As a result, Model IV was chosen as the best-fitting model for investigating the relationship between SBA and health insurance coverage among women.

### Fixed effect results for PNC

From Table 5, women covered by health insurance were more likely to utilise PNC (aOR=1.42, 95% CI 1.37 to 1.48). Women with some formal education were more likely to utilise PNC than those with no education. For instance, women who had secondary education were 42% more likely (aOR=1.42, 95% CI 1.38 to 1.47) to utilise PNC. The odds of utilising PNC were highest among women in their early 40s (aOR=1.32, 95% CI 1.24 to 1.40), those who were divorced (aOR=1.42, 95% CI 1.31 to 1.54) and African traditionalists (aOR=1.50, 95% CI 1.40 to 1.61). Also, women exposed to the mass media (aOR=1.58, 95% CI 1.55 to 1.62) had higher odds of utilising SBA. Conversely, multiparous women, those who had a major problem obtaining permission to utilise PNC (aOR=0.78, 95% CI 0.76 to 0.80) and resided in rural areas (aOR=0.87, 95% CI 0.84 to 0.89) had lower odds of utilising ANC. No discernible patterns were observed between wealth index, subregion and SBA utilisation.

### Random effect results for PNC

The results of Model 0 in Table 5 reveal that PNC varies significantly across the clusters in the study (σ2=0.312, 95% CI 0.275 to 0.354). Model 0 further showed that approximately 9% of the prevalence of PNC was linked to the between-cluster variations (ICC=0.087).

The between-cluster difference decreases further, from 8% in Model I to 7% in the model with both individual- and contextual-level variables (Model IV). This suggests that differences in the probability of attending PNC can be explained by variances across clusters. Furthermore, the AIC values confirmed a subsequent decrease, indicating that there is a significant improvement from the empty model (Model O) to the complete model (Model IV). This validates the final model's goodness of fit as determined by the analysis. As a result, Model IV was chosen to forecast the relationship between PNC and health insurance coverage among women.

## Discussion

This study examined the role of health insurance coverage in maternal healthcare utilisation among women of reproductive age in 28 SSA countries using DHS data. The overall prevalence of maternal healthcare utilisation was 58, 70.6 and 40.7% for ANC, SBA and PNC, respectively. Regarding health insurance coverage, we found that the overall prevalence was 6.4%. While Ghana recorded the highest coverage (66.8%), the lowest was recorded in Burkina Faso (0.4%). Women covered by health insurance were more likely to utilise ANC (aOR=1.48, 95% CI 1.41 to 1.54), SBA (aOR=1.37, 95% CI 1.30 to 1.45) and PNC (aOR=1.42, 95% CI 1.37 to 1.48) than those not covered.

The higher prevalence of SBA observed in this study compared with the other maternal health services (ANC and PNC) may be due to the efforts by the various governments in the SSA to achieve the global SDG 3.4 target of considerably reducing maternal mortality to <70 deaths per 100 000 live births by 2030^10^ through interventions that ensure that any woman expecting a child delivers at a certified health facility and with the assistance of a qualified health professional. Examples of such interventions include training more health professionals and their deployment to rural areas as part of the community-based health implementation, training and integration of traditional birth attendants into the health system to assist health professionals in the emergency deliveries, improve education among women, as well as increase the accessibility and affordability of maternal health services through various forms of health insurance schemes.^35,36^

The very low prevalence of health insurance coverage recorded in SSA, especially in countries like Burkina Faso, could be attributed to the myriad of challenges facing health insurance policy implementation in most of these countries, including poor funding, corruption, implementation challenges, high subscription and premium costs and the lack of political commitment to expand health insurance coverage to the pregnant women, indigents and informal sector workers who need the scheme the most.^37–44^ The abysmal health insurance coverage observed in the subregion inhibits progress towards achievement of UHC for at least 80% of their populace by 2030.^10^

Despite the overall low health insurance coverage recorded in SSA, countries like Ghana and Gabon recorded high coverage that could be attributed to the interventions put in place by those countries to ensure that their residents are covered. For instance, in Ghana, children aged <18 y, older people aged >70 y, indigents, formal sector workers who contribute to Social Security and National Insurance Trust (SSNIT) and SSNIT pensioners are exempt from paying the annual premiums required for membership of the scheme.^45–47^ In 2008, the country also incorporated a free maternal health policy into the National Health Insurance Scheme (NHIS), which makes ANC, SBA and PNC free for women. This, thus, encourages women of reproductive age, especially pregnant women, to subscribe more to the scheme.^45,46^ It is, therefore, not surprising that Ghana recorded the highest health insurance coverage of >70% prevalence of ANC, SBA and PNC utilisation. Linked closely to the high NHIS coverage and high maternal healthcare utilisation is the continuous decline in Ghana's maternal mortality ratio,^48^ which points to the effectiveness of the various interventions geared towards achieving the SDG targets of reducing maternal mortality and attainment of UHC.

As our main explanatory variable, we found that ownership of health insurance was an important predictor of maternal healthcare utilisation. Women covered by health insurance were more likely to utilise ANC, SBA and PNC than those who were not covered. This observation could be due to the financial protection health insurance offers to women during pregnancy, at childbirth and during the postnatal period. This finding is consistent with a previous study by Ameyaw et al.,^5^ which showed that health insurance serves as a cost-cutting and enabling intervention that enhances maternal healthcare utilisation, especially among women from the lower-income index. Out-of-pocket payments for maternal healthcare at the point of service delivery constitute the foremost barrier to maternal healthcare utilisation, especially among vulnerable and poor women in SSA.^12,13,49^ Hence, health insurance policies implemented in the subregion by the various governments exist as essential pro-poor initiatives intended to lift the financial barrier to maternal service utilisation among women.^34,50,51^ Our findings, where women with health insurance had higher odds of utilising maternal health services, shows that health insurance coverage could be a major fulcrum for SSA countries to achieve UHC and reduce maternal mortality to 70 per 100 000

live births by the end of 2030, as prescribed by the global SDG agenda 3.1 and 3.8 targets.^10,52^

Aside from health insurance coverage, we found other variables to be important predictors of maternal healthcare utilisation. For instance, we found a significant association between higher educational status and maternal health service utilisation. This observation agrees with previous studies that posited that women with low educational backgrounds are less likely to utilise maternal healthcare compared with educated women.^52,53^ This could be because women who are educated and empowered have increased decision-making power, freedom for making choices, informed choice and accept the responsibility for interventions (insurance subscription).^34,54,55^ This observation demonstrates the positive link between empowerment through education and informed decision making to utilise maternal healthcare.^5,56,57^

We found that maternal healthcare utilisation was associated with the wealth status of women. Women with stable financial capabilities were more likely to visit ANC during their pregnancy, have their births attended to by skilled professionals and access PNC.^55,58,59^ This observation points to poverty as a major barrier responsible for poor maternal healthcare utilisation among women. This could be explained by the fact that women who have financial power will be able to pay for transportation from their abodes to the health facility and be able to afford the maternal healthcare bills that result from other services that may not have health insurance coverage.^60,61^ On the contrary, their poor counterparts, especially those from rural settings, may be unable to afford transportation to the health facilities that are most remote from their residences, nor afford the other medical costs that may accumulate from the maternal services, as not all services are covered by health insurance in most countries in SSA.^62–64^ For example, even although maternal healthcare is free in Ghana under the national health insurance policy of 2003 (Act 650), the cost of transportation from the abodes of women to the remote health facilities where they receive maternal health services can be a barrier to dissuade poor women from constantly utilising maternal health services.^65–68^

Our study also found that women exposed to mass media have increased odds of utilising maternal healthcare. This finding is congruent to previous studies that have also shown that exposure to the mass media has a positive influence on the utilisation of ANC services among women.^69–73^ This observation could be explained by the fact that when women are exposed to mass media, they are then informed about the importance of maternal healthcare utilisation and the possible complications or consequences to the mother or child that can occur when not accessing maternal healthcare. Their exposure to mass media could also inform them regarding the benefit of health insurance or the services that are covered by national health insurance and free maternal healthcare policies.^71,72,74,75^

We noted in this study that multiparous women were less likely to utilise maternal healthcare. This finding is consistent with previous studies that also found a significant association between parity and maternal healthcare utilisation.^76–78^ This points to the fact that parity is an important predictor of adequate maternal healthcare utilisation, especially the antenatal services.^79,80^ This could be explained by the fact that multiparous women have the perceived confidence that they have more experience with pregnancy and childbirth, hence they are reluctant to utilise maternal health services, mostly the ANC services, during their subsequent pregnancies.^77,81,82^ It could also be due to any dissatisfaction they feel about the quality of previous care received or any negative attitudes displayed by health professionals they have experienced during their previous pregnancies.^83,84^

Concerning gaining permission from a spouse before utilising maternal healthcare, we found that women who have a major problem securing permission from their spouses have lower odds of utilising maternal health services compared with women who do not have a problem securing permission. This finding is consistent with previous studies that noted similar observations.^85,86^ This indicates that if husbands do not see any need for women to utilise maternal healthcare, then it becomes difficult for those women to utilise the necessary maternal healthcare, as going against the decision of their husbands may be perceived by those husbands and their families as insubordination, who hence refuse to provide money for transportation and other services.^76,87^ The observation that women sought consent from their partners before utilising maternal healthcare indicates a requirement to involve male partners in maternal healthcare utilisation and interventions, including education.^88^

Our findings revealed that the place of residence is significantly associated with maternal healthcare utilisation. Women from rural settings were less likely to utilise maternal healthcare. This finding corroborates previous studies^76,89–91^ and could be attributed to the limited number of facilities in rural areas. The distance from remote rural areas to major health facilities is also far, coupled with deplorable road networks. The difficulty in securing transportation and its associated costs could, therefore, deter rural women from utilising maternal healthcare.^77,92–94^

In Sierra Leone, while maternal healthcare utilisation was generally high, health insurance coverage was low. The implication, therefore, is that other variables (such as level of education, wealth status, exposure to mass media and place of residence), which have been proven influential in other studies, could have influenced the high utilisation levels.^34,53–55,60,61,76–78,89–91^ The high levels of maternal health utilisation in Sierra Leone could also have been due to interventions, including the implementation of a Free Health Care Initiative for pregnant and lactating mothers that started in April 2010.^95,96^ The policy exempts pregnant women and breastfeeding mothers from paying fees for services at the point of delivery.^95,96^

While we made some important findings, our study has several limitations. First, the cross-sectional nature of our data implied that we were unable to establish causality between health insurance coverage and maternal healthcare utilisation. Also, the self-reported nature of the responses of study participants in the DHS meant that there was the possibility of social desirability bias. In addition, the fact that some of the surveys used in this current study were conducted >5 y ago could affect the comparability of its findings with those of older surveys. Another limitation of this study may be that there is an overlap in reporting insurance in countries (like Ghana) where free maternal healthcare likely has a significant impact on healthcare utilisation, but we do not have a way to estimate the impact of this effect.

These limitations notwithstanding, a major strength of our analysis is the use of nationally representative data from 28 SSA countries that address the issue of the generalisability of our findings and conclusions. Our use of multilevel regression analyses also took into consideration the data collection procedure used in the DHS, ensuring robust analysis. Finally, our study is the first multicountry effort using the nationally representative DHS data to examine the influence of health insurance coverage on maternal healthcare utilisation in SSA. It, therefore, contributes extensively to the literature on health insurance coverage and maternal healthcare utilisation among women in the subregion.

## Conclusion

We found that health insurance coverage is an important predictor that could increase maternal healthcare utilisation among women in SSA. To speed up progress towards attainment of the SDG 3 targets related to the reduction of maternal mortality and achievement of UHC using health insurance, our study recommends that countries with low health insurance coverage (Burkina Faso, Benin, Chad and Gambia) need to adopt/step up interventions that have proven to be effective in other countries. Specific interventions include a reduction in premium costs for all potential subscribers and the exemption of women of reproductive age from the payment of such premiums for ANC, SBA and PNC.

## Supplementary Material

ihac017_Supplemental_File

## Data Availability

The datasets generated and/or analysed during the current study are freely available to the public at https://dhsprogram.com/data/available-datasets.cfm.
