# Supplementary material for: Towards achievement of Sustainable Development Goal 3: multilevel analyses of demographic and health survey data on health insurance coverage and maternal healthcare utilisation in sub-Saharan Africa
Source: Int Health. 2022 Apr 19;15(2):134–49. doi: 10.1093/inthealth/ihac017 (PMC9977256; doi:10.1093/inthealth/ihac017)
Supplement: ihac017_Supplemental_File [file ihac017_supplemental_file.docx]

| **Table S1. Description of sample** | | |  |
| --- | --- | --- | --- |
| **Countries** | **Year of survey** | **Weighted N** | **Weighted %** |
| Angola | 2015-16 | 8,516 | 4.35 |
| Burkina Faso | 2010 | 10,101 | 5.16 |
| Benin | 2017-18 | 9,095 | 4.65 |
| Burundi | 2016-17 | 8,992 | 4.6 |
| DR Congo | 2013-14 | 11,010 | 5.63 |
| Congo | 2011-12 | 5,886 | 3.01 |
| Cote d'Ivoire | 2011-12 | 5,207 | 2.66 |
| Cameroon | 2018 | 6,661 | 3.4 |
| Ethiopia | 2016 | 7,673 | 3.92 |
| Gabon | 2012 | 3,656 | 1.87 |
| Ghana | 2014 | 4,176 | 2.13 |
| Gambia | 2013 | 2,049 | 1.05 |
| Guinea | 2018 | 5,492 | 2.81 |
| Kenya | 2014 | 6,917 | 3.54 |
| Comoros | 2012 | 1,993 | 1.02 |
| Liberia | 2019-20 | 4,066 | 2.08 |
| Lesotho | 2014 | 2,595 | 1.33 |
| Mali | 2018 | 6,666 | 3.41 |
| Malawi | 2015-16 | 13,562 | 6.93 |
| Nigeria | 2018 | 22,041 | 11.27 |
| Namibia | 2013 | 3,797 | 1.94 |
| Sierra Leone | 2019 | 7,385 | 3.77 |
| Senegal | 2010-11 | 6,916 | 3.53 |
| Chad | 2014-15 | 3,687 | 1.88 |
| Togo | 2013-14 | 4,842 | 2.48 |
| Uganda | 2014-15 | 10,225 | 5.23 |
| Zambia | 2018 | 7,407 | 3.79 |
| Zimbabwe | 2015 | 5,038 | 2.57 |
| **All countries** |  | **195,651** | **100.00** |

DR Congo = Democratic Republic of Congo
